# A chain mediation model of inclusive leadership and voice behavior among university teachers: evidence from China

**DOI:** 10.1038/s41598-023-50018-2

**Published:** 2023-12-16

**Authors:** Chunlei Liu, Min Wu, Xiaoqin Chen

**Affiliations:** 1 School of Educational Science, Hunan Normal University, Changsha, 410081 Hunan People’s Republic of China; 2 School of International Education, Guangxi University of Science and Technology, Liuzhou, 545000 Guangxi People’s Republic of China; 3 School of Educational Science, Hunan University of Arts and Science, Changde, 415000 Hunan People’s Republic of China

**Keywords:** Psychology, Human behaviour

## Abstract

As a vital mode in which teachers can participate in university management, voice behavior is an important way of enhancing the efficiency of organizational decision-making, promoting democratic management, and facilitating sustainable development in universities. Although previous studies have confirmed the positive impact of inclusive leadership on employees' voice behavior, the mechanism underlying this effect remains unclear. Therefore, based on the cognitive-affective system theory of personality, this study aims to examine the mediating effects of psychological empowerment and organizational identification on the relationship between inclusive leadership and voice behavior among university teachers. A total of 517 valid questionnaires were administered to university teachers in mainland China using a convenience sampling approach. Structural equation modeling and bootstrap testing were used to analyze the data, and the results reveal that inclusive leadership is positively related to teachers’ promotive and prohibitive voice behavior. This relationship is mediated by psychological empowerment and organizational identification, in which context a partial mediating effect is observed in the relationship between inclusive leadership and promotive voice and a full mediating effect is observed in the relationship between inclusive leadership and prohibitive voice. These findings can enrich the extant research on the impact of inclusive leadership in the field of higher education to a certain extent. Moreover, they provide a new perspective that can support an in-depth analysis of the mechanism underlying the effect of inclusive leadership and generate valuable practical insights into ways of stimulating voice behavior among university teachers.

## Introduction

Due to the increasing development of the “double first-class” university construction program in China, the competition among Chinese universities has become increasingly fierce, thus making it crucial to enhance the internal governance ability of universities. Teachers, as the core stakeholders involved in university governance, play a crucial role in driving the development of universities. Their voice behavior is crucial to the implementation of democratic decision-making and management, the enhancement of organizational effectiveness and educational quality, and the promotion of the sustainable development of universities^1,2^. However, influenced by traditional Chinese Confucian culture, which is characterized by a high level of power distance, teachers' voice behavior is not common in practice, and their inclination to voice their opinions is not strong^3,4^. Most teachers choose to remain silent during organizational change, resulting in a waste of valuable human resources within university organizations^2^. Moreover, only limited research has investigated the voice behavior of teachers. Therefore, it is essential to explore ways of motivating university teachers to proactively engage in voice behavior and to provide more constructive advice and suggestions to their departments or colleges.

Previous research has indicated that various types of leadership behavior or leadership styles, such as transformational leadership^5^, authentic leadership^6^, ethical leadership^7^, and servant leadership^8^, are key contextual factors that influence teachers' voice behavior. However, due to the gradual flattening of organizational structures in universities, the breaking down of organizational boundaries, and the increasing global mobility of university teachers and researchers, traditional leadership styles have become less applicable. Inclusive management has emerged as a new paradigm for university governance^9^. Inclusive leadership, as a core aspect of inclusive management, represents a new leadership style in the contemporary economic and management context^10^, which emphasizes “openness, accessibility and availability” in interactions with subordinates^11^. Unlike other leadership styles, inclusive leadership is a core form of relational leadership^12^, which emphasizes respect, inclusion, and the provision of feedback from leaders to employees^13^. Given the high power distance that characterizes Chinese culture, such a leadership style that challenges the traditional image of authority is likely to have certain impacts on subordinates' attitudes and behaviors. As previous studies have found, inclusive leadership can increase employees’ innovation behavior^14^, reduce turnover intention^15^, and significantly improve organizational climate and performance^16^. However, whether the unique advantages of inclusive leadership can help promote teachers' voice behavior and the mechanisms underlying this impact remain unclear. Furthermore, previous studies on teachers' voice behavior have primarily focused on primary and secondary school teachers^17,18^, thereby neglecting the unique organizational context of higher education institutions and university teachers. Therefore, this study aims to explore the impact of inclusive leadership on teachers' voice behaviors and the underlying mechanisms by focusing on university teachers as the research population.

Research on the relationship between inclusive leadership and employees' voice behaviors has mainly been based on the perspectives of social exchange^19^, conservation of resources^20^, social identity^21,22^, causal attribution^23^, and basic need satisfaction^24,25^, and it has focused on the mediating role of variables such as leader-member exchange, caring ethical climate, organizational self-esteem, leader identification, and psychological safety. However, these studies have largely overlooked the importance of the motivational and emotional factors that drive employees’ voice behavior and the complexity of the employee-organization interaction, which extends beyond a merely contractual exchange^26^. The process of interaction between leaders and subordinates is influenced by a myriad of complex cognitive, motivational, and emotional factors^27^. Therefore, it may be difficult to systematically explain the impact of inclusive leadership on employees' voice behaviors from a single perspective, such as social exchange or social identity. According to the cognitive-affective system theory of personality (CASTP), inclusive leadership, as a significant contextual variable within an organization, is likely to indirectly influence individuals' voice behaviors by shaping their cognition and emotion^28^. Accordingly, this study introduces psychological empowerment and organizational identification as mediating variables.

Psychological empowerment refers to an individual’s internal and persistent motivation for work, which enables them to perceive themselves as empowered^29^. This perception is based on the individual’s comprehensive understanding of the meaning, competence, autonomy, and impact of their work^29^. Voice behavior, as a risky and challenging extrarole behavior, requires individuals to possess a strong motivation to exhibit autonomy and competence^30^. Psychological empowerment precisely satisfies individuals’ psychological needs for autonomy and competence^31^, thereby serving as a foundation for motivating individuals to voice their opinions proactively. Furthermore, inclusive leadership has been found to be positively associated with employees’ psychological empowerment^23^. As a result, psychological empowerment may serve as a mediator in the relationship between inclusive leadership and voice behavior among university teachers. Organizational identification refers to the cognitive process by which individuals perceive a sense of belonging to the organization and its members^32^. It refers to a crucial bond that connects individuals and organizations, which is characterized by emotional connections involving unity, honor, and disgrace^33^. This bond is based on the congruence of ideals, values, and behaviors^34^. Numerous studies have highlighted the significance of leaders’ support, care, and appreciation with regard to predicting employees’ organizational identification^34–38^. Inclusive leadership, as a positive leadership style, helps group members experience a sense of belonging in the context of work group while maintaining their uniqueness^39^. This leadership style focuses on establishing strong relationships between leaders and organization members. It is considered an important factor that affects individuals’ organizational identification^40^. Meanwhile, employees with high levels of organizational identification tend to view themselves as integral parts of the organization and to think and act from the organization's perspective^33^. Consequently, such employees are more likely to engage in extrarole behaviors, such as voice behavior^36,41^. The results of a meta-analysis concerning organizational identification have shown that this factor has a particularly strong predictive effect on employees’ extrarole behaviors, especially in a collectivist culture^33^. For this reason, organizational identification may mediate the relationship between inclusive leadership and university teachers’ voice behavior. In addition, previous research has demonstrated a positive association between psychological empowerment and organizational identification. Therefore, it is plausible that inclusive leadership may influence teachers’ voice behavior through the chain mediation of psychological empowerment and organizational identification.

In summary, based on the cognitive-affective system theory of personality, this study mainly focuses on the motivational aspect of voice behavior and aims to explore the mechanisms through which psychological empowerment and organizational identification mediate the relationship between inclusive leadership and university teachers’ voice behavior. Additionally, it strives to offer practical insights to promote democratic management within universities and contribute to the enrichment of inclusive leadership research.

### Inclusive leadership and voice behavior among university teachers

“Inclusiveness” is a relatively new concept in the field of organizational research, which was initially used mainly in the field of education, where scholars proposed the concept of "inclusive education" in response to the phenomena of diversity and differentiation in Western schools, advocating for the equal treatment of students who exhibited differences in terms of race, social status, religion, and other aspects^42^. It was not until 2006, when Nembhard and Edmondson introduced the idea of “inclusiveness” into leadership research, that the concept of inclusive leadership emerged^43^. Inclusive leadership refers to a leadership style in which leaders exhibit skill at listening to their subordinates’ viewpoints and appreciate their contributions^43,44^. As an effective leadership style among emerging leadership types, inclusive leadership emphasizes the establishment of good relationships during interactions with subordinates and encourages active organizational participation on the part of employees in response to leaders’ openness, accessibility, and availability^11^, ultimately fostering a supportive organizational environment for employees^45^. Numerous studies have shown that inclusive leadership can promote innovative behavior^46^, proactive behavior^47^, helping behavior^48^, and other organizational citizenship behaviors^49^ on the part of employees by enhancing their psychological safety^50^, organizational commitment^51^, work engagement^40^, and well-being^13^. Accordingly, this study predicts that inclusive leadership can also serve as a significant antecedent of teachers' voice behavior, which refers to a kind of extrarole interpersonal communication behavior in which members of an organization take the initiative to offer constructive ideas and opinions to those who have authority within the organization with the goal of improving their work or the status quo of the organization in a change-oriented manner^52^, rather than merely engaging in criticism^53^. Liang et al. categorized voice behavior into promotive voice, which focuses on offering suggestions, encouragement, and support to others and urging them to take positive actions or develop their potential, and prohibitive voice, which emphasizes providing early warnings and advice to prevent others from engaging in negative or harmful behaviors^54^. In the long term, voice behavior can enhance organizational effectiveness, but it may entail interpersonal risks for employees in the short term^30^.

According to social cognitive theory, individual behavior is influenced by situational stimuli^55^. In the university context, academic leaders play a crucial role in shaping the daily work environment of teachers and represent significant stimuli in the workplace. Therefore, the behavior or leadership style of university leaders affects the voice behavior of teachers, especially in the context of a relationship-oriented form of inclusive leadership^11^, which is characterized by an “openness” to management practices and explicitly sends a signal indicating that "suggestions are welcome " to their subordinates^39,56^. For example, Lee et al. found that inclusive leadership plays a vital role in positively predicting nurses' voice behaviors by enhancing their psychological safety^50^. Building on social exchange theory, Jiang et al. revealed that inclusive leadership positively influences employees’ voice behavior via leader-member exchange^19^. Relying on social identity theory, Guo et al.^22^ examined the effect of inclusive leadership on employees’ voice behavior through the mediation of leader identification. Thus, a positive and interactive work atmosphere within the university setting can be established by inclusive leaders who exhibit openness and encourage active participation in organizational decision-making and governance^57,58^. Moreover, inclusive leaders tend to dispel teachers’ misgivings by granting teachers a high degree of freedom to voice suggestions and encouraging them to express their true thoughts^51,58^. In particular, the accessibility and availability exhibit by inclusive leaders not only illustrate their exceptional leadership skills for teachers but also help establish good leader-member relationships and earn teachers’ trust^59^, which are key factors that encourage teachers to propose suggestions. Additionally, Randel noted that individuals’ belongingness and the degree to which they are valued for their uniqueness, which are enhanced by inclusive leadership, further motivate teachers to raise more constructive suggestions^39^. Therefore, the following hypotheses are proposed:

#### H1a

Inclusive leadership is positively related to university teachers’ promotive voice.

#### H1b

Inclusive leadership is positively related to university teachers’ prohibitive voice.

### The mediating role of psychological empowerment

Spreitzer defined psychological empowerment as an intrinsic form of motivation that reflects an individual’s positive orientation and sense of control toward work^29^, including the employee’s comprehensive perception of the meaning, competence, autonomy, and impact of that work^60^. Previous research has shown that inclusive organizational environments and leadership behavior can positively predict individuals' perceptions of psychological empowerment^14,61,62^. This positive psychological experience, in turn, can influence individuals’ behaviors and attitudes^18,23,63^.

On the one hand, inclusive leaders prioritize the involvement of subordinates in organizational management^43^, encouraging them to contribute new ideas^64^. This approach fosters trust and a sense of impact among teachers, thereby enhancing teachers' sense of empowerment at work. Moreover, according to Hollander, the effectiveness of inclusive leadership lies in its ability to empower subordinates and establish reciprocal leader-member relationships, thus increasing teachers’ sense of autonomy and responsibility^45^, satisfying their psychological need for self-determination and enhancing their psychological empowerment^11^. Additionally, leaders who exhibit inclusiveness create an environment of psychological safety, in which context teachers are supported in learning from their mistakes and understanding the expectations associated with their role^11^. This approach not only reduces teachers’ work anxiety and role stress but also deepens their perceptions of psychological empowerment.

On the other hand, voice behavior, although it is intended to enhance organizational effectiveness, is not without risk. By challenging the status quo or the authority of leadership^65^, individuals who engage in voice behavior may encounter potential negative influences. Therefore, for teachers who engage in voice behavior, their voice behavior must be rooted in strong perceptions of empowerment. Such empowerment serves as an essential motivation to engage in voice behavior, guiding teachers to weigh the effectiveness of their actions^66^. Parker et al. suggested that subordinates are more inclined to voice their suggestions or opinions when they believe that they possess the power and capability to do so^67^. Hence, teachers with strong perceptions of empowerment tend to exhibit greater autonomy and control over their work. They are more motivated to enhance their work efficiency by contributing new ideas and offering precautionary advice^63,68^. Additionally, teachers who exhibit a high sense of psychological empowerment also exhibit greater self-efficacy at work^61^. They firmly believe in the reasonableness and effectiveness of their voice. Consequently, these teachers are willing to contribute to the overall development of their organization. In contrast, teachers with weaker perceptions of empowerment are less likely to believe that they have a substantial impact on their department or college. This belief may lead them to feel less responsibility to improve the work atmosphere and consequently to exhibit weaker motivation to voice their opinions^69^. Therefore, we propose the following hypotheses:

#### H2a

Psychological empowerment plays a mediating role in the relationship between inclusive leadership and promotive voice among university teachers.

#### H2b

Psychological empowerment plays a mediating role in the relationship between inclusive leadership and prohibitive voice among university teachers.

### The mediating role of organizational identification

According to social identity theory, organizational identification refers to individuals’ psychological motivation to align their emotions, cognition, and behavior with their organization^34^. It refers to an individual’s self-definition as a member of the organization, resulting in a sense of belongingness and the integration of organizational values and goals into the individual’s own self-concept^33,70,71^. Previous studies have shown that organizational identification is a critical predictor of employees’ organizational citizenship behavior, such as voice behavior^36,38,72–74^. A higher level of organizational identification enables employees to develop a sense of ownership, consider issues from the perspective of organizations, and engage in behaviors that benefit organizations^75^. Conversely, when employees have a weaker sense of organizational identification, they often distance themselves from the organization, exhibit indifference toward its future and fate, and lack the motivation to engage in behaviors that are in the organization’s interest. Therefore, teachers with a strong sense of organizational identification exhibit a heightened sense of ownership and responsibility with regard to their work. They internalize the goals set by their leaders^76^, resulting in greater passion to contribute to the growth and advancement of their institution^38^. Consequently, such employees are more inclined to generate innovative ideas and offer valuable suggestions. Furthermore, research has indicated that individuals who possess a sense of organizational identification are more likely to establish positive interpersonal relationships and emotional connections with their immediate leaders and colleagues^76^. This enhanced integration within the organization serves as a cohesive force, decreasing teachers' perception of risk in voicing their opinions. Thus, they become more courageous with regard to addressing organizational issues and expressing their genuine thoughts.

Previous research has confirmed that communication is a prerequisite for organizational identification and that the communication climate has a greater impact on organizational identification than does communication content^77^. Inclusive leadership, which involves enhancing subordinates’ sense of belonging and valuing their uniqueness^64^, can foster a supportive communication climate that helps promote the emotional connections among organization members, thus enhancing individuals’ organizational identification^78^. Moreover, inclusive leadership emphasizes welcoming and appreciating employees’ contributions^11^. Research has already confirmed that leaders’ support and recognition have significant positive impacts on employee organizational identification^79^. Furthermore, Morgan demonstrated that the quality of the relationship between leaders and employees is a key factor with regard to organizational identification^80^. Inclusive leadership, as a distinct manifestation of relationship-oriented leadership, places a significant emphasis on the cultivation of strong relationships between leaders and members. Hence, it inevitably becomes one of the crucial determinants of individuals’ organizational identification. Additionally, inclusive leadership establishes a conducive environment for error management^14^, in which context teachers’ mistakes are not merely accepted but tolerated, thus contributing to the enhancement of teachers' sense of psychological safety and belonging. Therefore, we propose the following hypotheses:

#### H3a

Organizational identification mediates the relationship between inclusive leadership and promotive voice among university teachers.

#### H3b

Organizational identification mediates the relationship between inclusive leadership and prohibitive voice among university teachers.

### The chain mediating effects of psychological empowerment and organizational identification

Social identity theory suggests that organizational identification serves as a lasting emotional bond between employees and their organizations. This identification is influenced by both external contextual factors and internal cognitive processes^81^. Therefore, it is not surprising to observe varying degrees of organizational identification within the same organizational setting. In particular, employees’ perceptions of psychological empowerment play a crucial role in influencing their level of organizational identification. Employees who exhibit higher levels of psychological empowerment tend to perceive greater organizational support as well as a higher degree of value and importance with regard to their organizations. Moreover, they experience greater job autonomy and possess a higher sense of self-efficacy^82^. According to self-determination theory, the fulfillment of employees’ psychological needs for autonomy, competence, and relatedness in the workplace leads to a greater sense of belonging^83^. Consequently, they are more likely to exhibit greater dedication and actively work toward the achievement of organizational objectives. In return for the support provided by the organization, employees contribute their suggestions and personal efforts to promote the organization's growth and development. In essence, psychological empowerment fosters the development of organizational identification through a reciprocal process^84^. Therefore, the following hypotheses are proposed:

#### H4a

Teachers’ psychological empowerment is positively related to their organizational identification.

#### H4b

Psychological empowerment and organizational identification have a chain mediating effect on the relationship between inclusive leadership and promotive voice among university teachers.

#### H4c

Psychological empowerment and organizational identification have a chain mediating effect on the relationship between inclusive leadership and prohibitive voice among university teachers.

**Figure 1:**
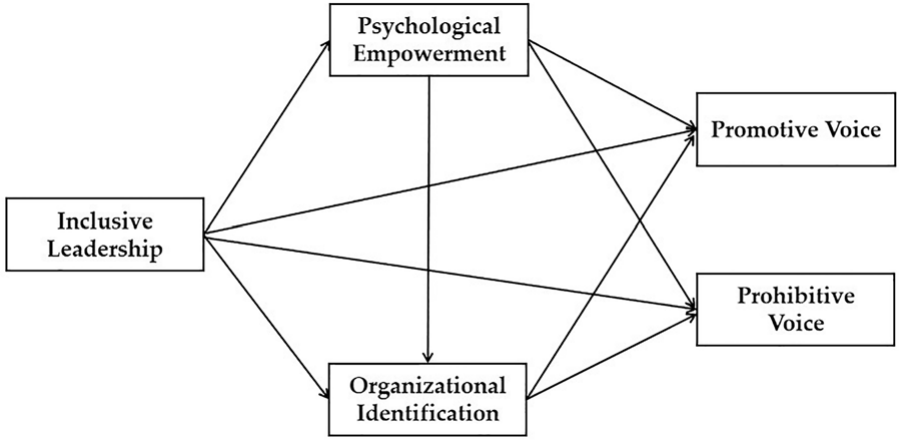
Proposed model.

In summary, we constructed a chain mediating model, as shown in Fig. 1.

## Materials and methods

### Participants and procedure

In this study, we first imported the prepared questionnaire into the Questionnaire Star platform to generate an electronic questionnaire. The electronic questionnaires were distributed to teachers who were currently working in colleges or universities in mainland China using the convenience sampling and snowball sampling methods via social platforms such as WeChat or QQ groups. Data were collected from June 2022 to July 2022. A total of 538 teachers from 35 universities participated in this survey voluntarily and anonymously. After eliminating invalid questionnaires that contained missing or contradictory information, 517 valid questionnaires were ultimately obtained, for an effective recovery rate of 96%. The demographic information of the valid sample is displayed in Table 1.

**Table 1:** Respondents’ profiles (n = 517).

Item	Category	Percentage (%)	Item	Category	Percentage (%)
Gender	Male	44.7	Degree received	Bachelor’s	20.3
	Female	55.3		Master’s	41.2
Age	≤ 35	36.2		Doctoral degree	38.5
	36–45	32.1	Institution level	Vocational college	28.6
	46–55	20.9		“Nondouble first-class” university	41.8
	≥ 56	10.8		“Double first-class” university	29.6
Academic title	Assistant	17.6	Teaching experience (in years)	≤ 3	19.3
	Lecturer	30.6		4–10	35.2
	Associate Prof	32.3		11–20	30.4
	Professor	19.5		≥ 21	15.1

### Measures

The questionnaire used in the current study consisted of two main parts, namely, a basic information section and a scale questions section, which included the inclusive leadership scale, the voice behavior scale, the psychological empowerment scale, and the organizational identification scale. To ensure the validity of the study, the scales were derived from mature scales developed by Western scholars. The four English-language versions of the scales were translated into Chinese using a translation and back-translation procedure. Without distorting the questionnaire, appropriate modifications were made to the wording and language of the original scales to ensure that the questionnaire used in this survey was in accordance with Chinese linguistic habits. All scale questions were scored on a five-point Likert scale ranging from 1 (strongly disagree) to 5 (strongly agree). Higher numbers indicated higher levels of agreement.

Teachers’ voice behavior was measured using a 10-item scale proposed by Liang et al.^54^, which included 5 items for promotive voice and 5 items for prohibitive voice, such as “I will actively voice my viewpoints that facilitate the advancement of my department or college” (promotive voice) and “I dare to express my opinion on issues that influence the performance of my department or college even though it may embarrass others” (prohibitive voice). The confirmatory factor analysis (CFA) indicated the good structural validity of this scale (X^2^/DF = 1.606, RMR = 0.025, RMSEA = 0.034, GFI = 0.979, IFI = 0.989, NFI = 0.971, TLI = 0.985, CFI = 0.989). The Cronbach's α coefficients of promotive voice and prohibitive voice in this study were 0.831 and 0.843, respectively.

Inclusive leadership was measured using a 9-item scale proposed by Carmeli et al.^11^, which included three subdimensions. Sample items included “My immediate leaders are willing to consider new ideas or suggestions from teachers” (openness), “I can always ask my leader for advice if I have questions” (accessibility), and “I can find my leader to discuss new problems that arise in my job” (availability). The CFA indicated the good structural validity of the scale (X^2^/DF = 1.899, RMR = 0.019, RMSEA = 0.019, GFI = 0.988, IFI = 0.997, NFI = 0.982, TLI = 0.996, CFI = 0.997). The Cronbach's α coefficient of this scale in this study was 0.868.

Psychological empowerment was measured using a 12-item scale constructed by Spreitzer^29^, which included the four dimensions of competence, meaning, self-determination, and impact. Example items included “The work I do means a lot to me”, “I have enough confidence to do my job”, “I can make my own decisions about how to do my work”, and “I have some impact on what happens in my department or college”. The CFA indicated the good structural validity of the scale (X^2^/DF = 1.128, RMR = 0.017, RMSEA = 0.016, GFI = 0.982, IFI = 0.998, NFI = 0.981, TLI = 0.997, CFI = 0.998). The Cronbach's α coefficient of this scale in this study was 0.907.

Organizational identification was measured using the unidimensional 6-item scale proposed by Mael et al.^32^. Example items included “The success of my department or college is my success” and “I would be embarrassed if media reports were to criticize my department or college”. The CFA indicated the good structural validity of the scale (X^2^/DF = 1.486, RMR = 0.014; RMSEA = 0.031, GFI = 0.992, IFI = 0.994, NFI = 0.989, TLI = 0.994, CFI = 0.996). The Cronbach's α coefficient of this scale in this study was 0.860.

Control variables: in this study, teachers’ personal characteristics, such as gender, age, education level, academic title, teaching experience, and university level (see Table 1), were included as control variables according to the recommendations of previous studies^65^.

### Data analysis

In this study, reliability and validity testing, descriptive statistics, correlation analysis, and common method bias testing were conducted using SPSS 26.0 software. Additionally, confirmatory factor analysis (CFA) and structural equation model path analysis were performed using AMOS 26.0 software. The significance of the mediating effect was tested using the bootstrap method, with 5000 repeated samplings. The results were further validated by estimating 95% bias-corrected bootstrap confidence intervals (CIs).

### Ethics statement

The study was conducted in accordance with the Declaration of Helsinki and approved by the Human Research Ethics Committee of Hunan Normal University. All participants agreed to participate in this research voluntarily; they provided informed consent when they completed the survey and were able to withdraw from the study freely at any time. In addition, our data were anonymized to ensure the privacy of all participants.

## Results

### Common method bias testing

Given that data were obtained from teachers’ self-reports, common method bias may have been present. To mitigate this possibility, an anonymous questionnaire survey was utilized in this study. Subsequently, exploratory factor analysis was conducted on all scale items using Harman’s single-factor test. The results indicated six factors with an eigenvalue greater than one, with the first factor explaining only approximately 26.72% of the variance, i.e., significantly below the threshold of 40%. Additionally, the results of the CFA conducted to investigate the one-factor model in Table 2 indicated that the model exhibited a poor fit (X^2^/DF = 11.078, RMSEA = 0.140, IFI = 0.542, TLI = 0.494, CFI = 0.540, GFI = 0.609). These findings indicate that common method bias was not a serious concern in the current study^85^.

**Table 2:** Results of the confirmatory factor analysis of the proposed model and the competing models (N = 517).

Measurement	X^2^/DF	RMSEA	SRMR	GFI	IFI	CFI	TLI	NFI
Five factor model (IL;PE;OI;PV;PHV)	1.342	0.026	0.026	0.953	0.985	0.985	0.983	0.944
Four factor model (IL;PE;OI;PV + PHV)	4.487	0.082	0.074	0.793	0.846	0.845	0.825	0.810
Three factor model (IL;PE + OI;PV + PHV)	7.674	0.114	0.085	0.679	0.771	0.699	0.665	0.671
Two factor model (IL;PE + OI + PV + PHV)	8.990	0.124	0.090	0.651	0.638	0.637	0.599	0.611
One factor model (IL + PE + OI + PV + PHV)	11.078	0.140	0.095	0.609	0.542	0.540	0.494	0.518

IL: Inclusive Leadership; PE: Psychological Empowerment; OI: Organizational Identification; PV: Promotive Voice; PHV: Prohibitive Voice.

In addition, as illustrated in Table 2, the fit index of the proposed five-factor model (X^2^/DF = 11.078, RMSEA = 0.140, IFI = 0.542, TLI = 0.494, CFI = 0.540, GFI = 0.609) was much better than that of the other four alternative models, thus indicating good discriminant validity among the five constructs.

### Reliability and validity testing

As shown in Table 3, each variable exhibited satisfactory reliability, with Cronbach’s alpha coefficients exceeding 0.70 and composite reliability (CR) values above 0.8. Moreover, the standard factor loadings of each scale ranged from 0.652 to 0.803, thus surpassing the 0.60 threshold. Furthermore, the average variance extracted (AVE) for each variable, which ranged from 0.50 to 0.63 and thus exceeded the 0.50 threshold, indicated that the scales exhibited good convergent validity. Additionally, the square root of the AVE for each variable was greater than the Pearson correlation coefficient between variables (Table 4), thus indicating good discriminant validity among the five constructs once again.

**Table 3:** Reliability and validity testing results (*N* = 517).

Variables	Items	Means	SD	Factor Loading	*P* value	Cronbach’s α	CR	AVE
Inclusive Leadership	IL1	3.56	0.905	0.654	< 0.001	0.868	0.84	0.63
	IL2	3.62	0.933	0.700	< 0.001			
	IL3	3.50	0.974	0.619	< 0.001			
	IL4	3.60	1.015	0.653	< 0.001			
	IL5	3.65	0.981	0.617	< 0.001			
	IL6	3.67	0.975	0.749	< 0.001			
	IL7	3.69	0.945	0.718	< 0.001			
	IL8	3.70	0.995	0.731	< 0.001			
	IL9	3.71	0.986	0.729	< 0.001			
Psychological Empowerment	PE1	3.76	0.927	0.771	< 0.001	0.907	0.87	0.58
	PE2	3.75	0.917	0.754	< 0.001			
	PE3	3.74	0.876	0.712	< 0.001			
	PE4	3.80	0.919	0.714	< 0.001			
	PE5	3.77	0.854	0.694	< 0.001			
	PE6	3.72	0.861	0.722	< 0.001			
	PE7	3.63	0.895	0.766	< 0.001			
	PE8	3.66	0.874	0.742	< 0.001			
	PE9	3.56	0.871	0.744	< 0.001			
	PE10	3.50	0.906	0.773	< 0.001			
	PE11	3.34	0.964	0.803	< 0.001			
	PE12	3.39	0.951	0.794	< 0.001			
Organizational Identification	OI1	3.40	0.915	0.696	< 0.001	0.860	0.86	0.50
	OI2	3.39	0.880	0.748	< 0.001			
	OI3	3.62	0.932	0.687	< 0.001			
	OI4	3.45	0.889	0.702	< 0.001			
	OI5	3.54	0.911	0.761	< 0.001			
	OI6	3.46	0.924	0.679	< 0.001			
Promotive Voice	PV1	3.73	0.923	0.727	< 0.001	0.831	0.83	0.50
	PV2	3.67	0.967	0.702	< 0.001			
	PV3	3.63	0.993	0.652	< 0.001			
	PV4	3.65	0.996	0.725	< 0.001			
	PV5	3.70	0.959	0.717	< 0.001			
Prohibitive Voice	PHV1	3.52	0.962	0.699	< 0.001	0.843	0.84	0.52
	PHV2	3.56	0.906	0.739	< 0.001			
	PHV3	3.43	0.935	0.697	< 0.001			
	PHV4	3.44	0.958	0.790	< 0.001			
	PHV5	3.42	0.960	0.678	< 0.001			

CR: composite reliability; AVE: average variance extracted.

**Table 4:** Descriptive statistics and intercorrelations (*N* = 517).

Variables	Means	SD	1	2	3	4	5
Inclusive Leadership	3.63	0.67	**0.79**				
Psychological Empowerment	3.64	0.63	0.382***	**0.76**			
Organizational Identification	3.48	0.70	0.257***	0.365***	**0.71**		
Promotive Voice	3.68	0.75	0.299***	0.383***	0.264***	**0.71**	
Prohibitive Voice	3.47	0.74	0.244***	0.379***	0.538***	0.298**	**0.72**

**p < 0.01, ***p < 0.001 (two-tailed); The bold number is the square root of the AVE.

### Descriptive statistics and correlation analysis

The means, standard deviations, and correlation coefficients among the variables are shown in Table 4. The results revealed that inclusive leadership was positively correlated with psychological empowerment (β = 0.382, *p* < 0.001), organizational identification (β = 0.257, *p* < 0.001), promotive voice (β = 0.299, *p* < 0.001), and prohibitive voice (β = 0.382, *p* < 0.001). Psychological empowerment was positively correlated with organizational identification (β = 0.365, *p* < 0.001), promotive voice (β = 0.383, *p* < 0.001), and prohibitive voice (β = 0.379, p < 0.001). Organizational identification was positively correlated with promotive voice (β = 0.264, *p* < 0.001) and prohibitive voice (β = 0.538, *p* < 0.001). Promotive voice was positively correlated with prohibitive voice (β = 0.298, *p* < 0.01).

### Hypothesis testing

**Figure 2:**
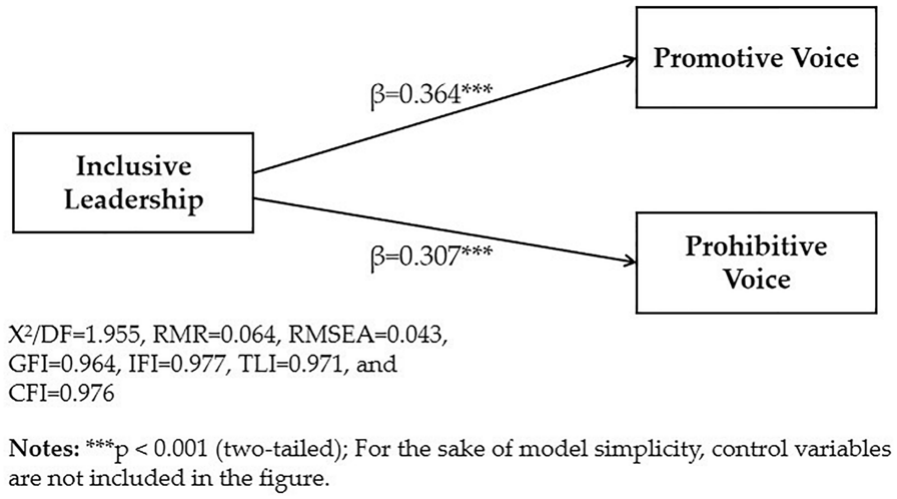
Structural equation model output without mediation.

To examine the direct impact of inclusive leadership on promotive voice and prohibitive voice among university teachers, a direct path model was first developed, in which context gender, age, education level, academic title, teaching years, and institution level were included as controls. The model indicated a good fit to the data (X^2^/DF = 1.955, RMR = 0.064, RMSEA = 0.043, GFI = 0.964, IFI = 0.977, TLI = 0.971, and CFI = 0.976). The results, which are displayed in Fig. 2, revealed that inclusive leadership had significant positive effects on university teachers’ promotive voice (β = 0.364, *p* < 0.001) and prohibitive voice (β = 0.307, *p* < 0.001). Thus, both H1a and H1b were statistically supported.

**Figure 3:**
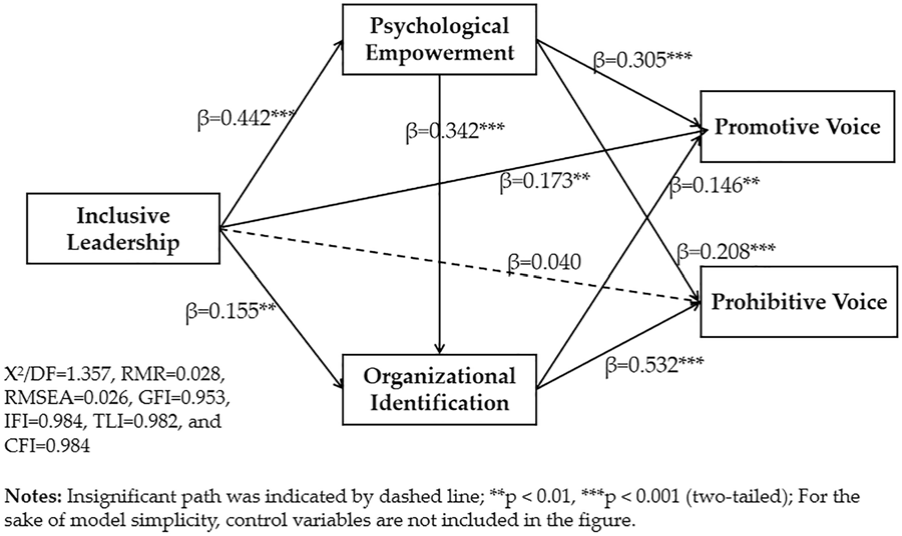
Structural equation model output with mediation.

Next, the chain mediation model was tested, and the fit of the model was good (X^2^/DF = 1.357, RMR = 0.028, RMSEA = 0.026, GFI = 0.953, IFI = 0.984, TLI = 0.982, and CFI = 0.984). As illustrated in Fig. 3, all the standard direct path coefficients in the proposed model were significant, with the exception of the direct path from inclusive leadership to teachers’ prohibitive voice (β = 0.040, *p* = 0.426 > 0.05). First, inclusive leadership had a significant and positive impact on university teachers’ promotive voice behavior (β = 0.173, *p* < 0.01) when controlling for the mediating variables. Second, inclusive leadership had a significant and positive influence on psychological empowerment (β = 0.442, *p* < 0.001) and organizational identification (β = 0.155, *p* < 0.01). Psychological empowerment was also found to have significant effects on teachers’ promotive voice (β = 0.305, *p* < 0.001) and prohibitive voice (β = 0.208, *p* < 0.001). Furthermore, organizational identification significantly predicted teachers’ promotive and prohibitive voice (β = 0.146, *p* < 0.01; β = 0.532, *p* < 0.001). In addition, teachers’ perceptions of empowerment significantly predicted their organizational identification (β = 0.342, *p* < 0.001); thus, H4a was statistically supported.

To test the mediating effects of psychological empowerment and organizational identification on the relationship between inclusive leadership and teachers’ voice behavior, this study used a bias-corrected nonparametric percentile bootstrap method with 5,000 random replicates of the sample (n = 517); a 95% confidence interval was also estimated. The results are shown in Table 5.

**Table 5:** Results of the mediation analysis (*N* = 517).

	Effect value	Standard errors	95% Confidence intervals	Effect size (%)
*Outcome variable: Promotive voice*				
Total effect	0.353	0.054	[0.245, 0.453]	
Direct effect	0.173	0.062	[0.054, 0.296]	49.01
Total indirect effect	0.180	0.034	[0.120, 0.254]	50.99
Path 1: Inclusive leadership → Psychological empowerment → Promotive voice	0.135	0.043	[0.100, 0.199]	38.24
Path 2: Inclusive leadership → Organizational identification → Promotive voice	0.023	0.017	[0.005, 0.076]	6.52
Path 3: Inclusive leadership → Psychological empowerment → Organizational identification → Promotive voice	0.022	0.013	[0.008, 0.059]	6.23
*Outcome variable: Prohibitive voice*				
Total effect	0.294	0.053	[0.186, 0.395]	
Direct effect	0.040	0.054	[− 0.066, 0.148]	13.61
Total indirect effect	0.254	0.042	[0.175, 0.339]	86.39
Path 1: Inclusive leadership → Psychological empowerment → Prohibitive voice	0.092	0.037	[0.054, 0.198]	31.29
Path 2: Inclusive leadership → Organizational identification → Prohibitive voice	0.082	0.048	[0.022, 0.211]	27.89
Path 3: Inclusive leadership → Psychological empowerment → Organizational identification → Prohibitive voice	0.080	0.029	[0.058, 0.172]	27.21

First, in the analysis that included promotive voice as the outcome variable, the mediating effect of psychological empowerment on the relationship between inclusive leadership and university teachers’ promotive voice was significant (the mediating effect value was 0.135, *p* < 0.001, 95% CI [0.100, 0.199]), with an effect size of 38.24%; thus, H2a was supported statistically. The mediating effect of organizational identification on the relationship between inclusive leadership and university teachers’ promotive voice was significant (the mediating effect value was 0.023, *p* < 0.01, 95% CI [0.005, 0.076]), with an effect size of 6.52%; thus, H3a was also supported statistically. Finally, the chain mediating effect of psychological empowerment and organizational identification on the relationship between inclusive leadership and university teachers’ promotive voice was also significant (the chain mediating effect value was 0.022, *p* < 0.01, 95% CI [0.008, 0.059]), with an effect size of 6.23%; thus, H4b was also supported statistically. The total mediating effect value of the three mediated paths was 0.180, accounting for 50.99% of the total effect (0.353).

Furthermore, in the analysis including prohibitive voice as the outcome variable, the direct effect of inclusive leadership on prohibitive voice was nonsignificant (β = 0.040, p > 0.05); thus, psychological empowerment and organizational identification fully mediated the relationship between these two factors. Specifically, the mediating effect of psychological empowerment on the relationship between inclusive leadership and university teachers’ prohibitive voice was significant (the mediating effect value was 0.092, *p* < 0.001, 95% CI [0.054, 0.198]), with an effect size of 31.29%; H2b was thus supported statistically. The mediating effect of organizational identification on the relationship between inclusive leadership and prohibitive voice was significant (the mediating effect value was 0.082, *p* < 0.001, 95% CI [0.022, 0.211]), with an effect size of 27.89%; thus, H3b was also supported statistically. Finally; the chain mediating effect of psychological empowerment and organizational identification on the relationship between inclusive leadership and prohibitive voice was also significant (the chain mediating effect value was 0.080, *p* < 0.001, 95% CI [0.058, 0.172]), with an effect size of 27.21%; thus, H4c was also supported statistically. The total mediating effect value of the three mediated paths was 0.254, accounting for 86.39% of the total effect (0.294).

## Discussion

### The effect of inclusive leadership on university teachers’ voice behavior

The findings of this study indicated that the total effects of inclusive leadership on teachers’ promotive voice and prohibitive voice behavior are significant, thus demonstrating that inclusive leadership style, as a form of positive organizational support, influences teachers’ extrarole behavior^19,86,87^. These results are consistent with the findings of previous research^21,22,24^. This finding indicates that when university leaders are open to adopting teachers' suggestions, teachers are more likely to provide positive feedback, as they feel trusted and valued by their leaders^20^. Moreover, when leaders are accessible and able to provide guidance and resources to teachers, the likelihood of proactive extrarole behavior on the part of teachers is further enhanced^49,88^. However, it is worth noting that although this study found that inclusive leadership has a significant direct impact on promotive voice among teachers, its direct effect on prohibitive voice is not significant. One possible explanation for this discrepancy is that prohibitive voice, which is characterized by raising precautionary concerns to prevent problems, is often accompanied by interpersonal risks and challenges the current status quo. However, whether to give advice is a rational choice made by teachers after weighing the potential benefits and potential risks of such prohibitive advice. Only when the potential benefits outweigh the potential risks are teachers likely to express their opinions and suggestions. In addition, this finding can be explained effectively by the two factor theory proposed by Fredrick Herzberg. In fact, both preventive and motivating factors are involved in motivating teachers to engage in prohibitive voice. Inclusive leadership, as an external contextual factor, acts as a preventive factor with regard to prohibitive voice. Namely, the absence of inclusive leadership restrains teachers from engaging in prohibitive voice. However, the presence of this factor does not guarantee that teachers automatically engage in prohibitive voice.

### The mediating effects of psychological empowerment and organizational identification

The current study revealed that psychological empowerment is an essential mediator in the relationship between inclusive leadership and teachers’ voice behaviors, with mediating effect sizes of 38.24% (when promotive voice is included as the outcome variable) and 31.29% (when prohibitive voice is included as the outcome variable). These findings are consistent with the results of previous research^23^ and can be explained by reference to self-determination theory^89^. Paolillo et al. found that inclusive leadership, as a supportive external context, can meet an individual’s basic psychological needs for competence, autonomy, and relatedness, thereby enhancing their motivation to engage in voice behavior^90^. In a high power distance context such as China, leadership behaviors and attitudes have significant impacts on subordinates' affection, cognition, and behaviors at work. Specifically, the openness, availability, and accessibility exhibited by university leaders can positively influence teachers’ perceptions of their competence and autonomy as well as the meaningfulness of their work^14,61^. As a result, this approach can contribute to the enhancement of their psychological empowerment^14^. The improvement of teachers' perceptions of psychological empowerment can further enhance their autonomy, sense of efficacy, and sense of meaning in work, thereby enabling them to identify problems in their work and promptly present suggestions and opinions to the relevant leaders^6^.

This study also found that organizational identification plays a mediating role in the relationship between inclusive leadership and voice behavior among university teachers, with mediating effect sizes of 6.52% (when promotive voice is included as the outcome variable) and 27.89% (when prohibitive voice is included as the outcome variable). According to social exchange theory, employees’ extrarole behavior is based on the benefits they receive from the organization, such as inclusive leadership and resource support, and the subsequent "equivalent" contributions they make in return^38^. Organizational identification plays a critical role in facilitating this reciprocal process^72^. The support and benefits provided by inclusive leadership can lead to perceptions of an “insider” identity and a sense of belonging among teachers, thereby leading to more positive attitudes toward and identification with the organization^91^. Furthermore, teachers with strong perceptions of organizational identification tend to possess a greater sense of responsibility, mission, and ownership at work^92^. Consequently, this approach leads to a higher motivation to engage in organizational citizenship behavior, especially when teachers encounter problems that may hinder the achievement of organizational goals.

Additionally, this study revealed that perceptions of psychological empowerment and organizational identification have a chain mediating effect on the relationship between inclusive leadership and university teachers’ voice behavior, which is consistent with the hypothesis proposed by the cognitive-affective system theory of personality. According to this theory, each individual possesses a unique cognitive and affective system, which interacts with the social environment to produce individual-specific patterns of behavior^28^. Inclusive leadership, as a supportive organizational context, not only has a direct impact on teachers’ voice behavior but also further influences their behavioral choices by affecting their cognitive and affective systems. Psychological empowerment refers to teachers’ comprehensive psychological cognition of their work meaning, competence, autonomy, and impact, which is based on their assessment of the inclusive work environment^29^. Teachers with strong perceptions of empowerment exhibit a stronger sense of emotional belonging with regard to their organization^82^ and are thus more inclined to engage in organizational citizenship behavior in return for inclusive leaders’ support and care^93^. Especially in the analysis that included teachers’ prohibitive voice as the outcome variable, psychological empowerment and organizational identification played full chain mediating roles, with a chain mediation effect size of 27.21%. This finding suggests that individual internal cognition (psychological empowerment) and emotional experience (organizational identification) are the primary factors that motivate teachers to express their opinions rather than inclusive external organizational contextual factors. These findings make significant contributions to the theoretical foundation of human resources management in higher education organizations.

### Practical implications

As the main channel for university teachers’ participation in the democratic management of universities, the effective role such teachers’ voice behavior is conducive to the autonomous identification and resolution of issues as well as the improvement of the overall level of education. The findings of this research have some practical implications regarding ways of stimulating university teachers' voice behavior. First, universities should aim to enhance leaders’ inclusiveness and establish an organizational culture and environment that fosters constructive suggestions. This goal can be achieved by implementing inclusive leadership with the goals of promoting an open and inclusive working atmosphere, caring for the needs of teachers, providing readily available support and assistance, and offering opportunities and channels for teachers to express their opinions and suggestions. This approach can alleviate teachers’ anxiety and concerns about exercising a prohibitive voice. Second, this study found that inclusive leadership enhances teachers' perception of psychological empowerment, which in turn leads to increased voice behavior. Therefore, it is crucial for university leaders to foster a sense of meaning, competence, self-determination, and impact among teachers within the organization. This goal can be achieved by encouraging teachers' active participation and integration in organizational affairs, providing them with recognition, support, and respect, and promoting their positive psychological well-being within the organization. As a result, teachers are motivated to actively engage in voice behaviors that contribute to organizational development. Additionally, organizational identification is an important mediator in the relationship between inclusive leadership and teachers' voice behavior. Therefore, it is suggested that higher education administrators should take measures to enhance teachers' organizational identification, such as by expressing concern for their professional development, helping them solve the difficulties they encounter at work and in life, and fostering a harmonious and friendly work environment. These measures can effectively promote teachers' voice behavior.

### Limitations

Although this study reveals certain insights, some limitations should also be taken into consideration. First, the present study utilizes cross-sectional data, which makes it difficult to establish cause-and-effect relationships among the variables. Future research should employ longitudinal designs to further validate this proposed model. Second, this study focuses exclusively on the mediating roles of psychological empowerment and organizational identification, thereby neglecting the influence of other psychological cognitive factors. To fully explore the mediating mechanisms underlying the effects of inclusive leadership on teachers’ voice behavior, future studies should continue to explore other mediating pathways. In addition, no moderating variables were considered in this study, and future research could consider teachers’ personal characteristics as potential moderating variables to enhance our understanding of the relationship between inclusive leadership and teachers' voice.

## Conclusions

Based on the cognitive-affective system theory of personality (CASTP), this study explored the influence of inclusive leadership on voice behavior among university teachers and the underlying mechanisms. An analysis of 517 questionnaires collected from university teachers revealed that inclusive leadership has a positive effect on the voice behavior of university teachers. Perceptions of psychological empowerment and organizational identification both play crucial roles as individual mediators and sequential mediators of the relationship between inclusive leadership and teachers' voice behavior. Specifically, perceived psychological empowerment and organizational identification partially mediate the relationship between inclusive leadership and promotive voice and fully mediate the relationship between inclusive leadership and prohibitive voice. The findings have practical implications regarding the enhancement of leaders’ inclusiveness and the establishment of an organizational culture that fosters teachers’ psychological empowerment and organizational identification with the goal of motivating their voice behaviors.

## Data Availability

The data are available from the corresponding author upon request.
